# Serum cytokines and tissue doppler imaging as markers of prognosis of chronic myocarditis due to chagas disease

**DOI:** 10.1016/j.bjid.2026.105786

**Published:** 2026-02-21

**Authors:** Ivan Santana Batista Soares, Edmundo Câmara, Manoel Barral-Netto, Carlos Rico Quintero, Benelson Guimarães Carvalho, Fábio Bulhões, Alex Cleber Improta-Caria, Roque Aras-Júnior

**Affiliations:** a Faculty of Medicine, Graduate Program in Medicine and Health (PPGMS), Salvador, BA, Brazil; b Fundação Oswaldo Cruz (FIOCRUZ), Instituto Gonçalo Moniz, Salvador, BA, Brazil; c Climecar, Cardiology Clinic, Itaberaba, BA, Brazil; d Universidade de São Paulo (USP), Laboratory of Biochemistry and Molecular Biology of the Exercise, São Paulo, SP, Brazil

**Keywords:** Chagas disease, Cytokines, Interleukins, T. cruzi, Tissue Doppler imaging

## Abstract

**Introduction:**

Insidious myocarditis due to Chagas disease evolves with low inflammatory potential. Determining biomarkers can help find individuals at risk for Heart Failure (HF) or arrhythmias. This study aimed to evaluate the role of serum cytokines and myocardial tissue Doppler imaging as prognostic markers of Chagas myocarditis.

**Methods:**

This longitudinal study was carried out in a prospective cohort in which adults living with Chagas disease who had no HF were followed up for 10-years. Tissue Doppler imaging was performed, and inflammatory cytokines were measured. Kaplan-Meier survival analysis, unadjusted and adjusted survival analyses for some variables by Cox regression and multivariate binary logistic regression analysis were performed for the primary and total outcomes.

**Results:**

This study found that IL1-B and IL-13 were associated with primary and total events (*p* = 0.015 and *p* = 0.013 and *p* = 0.013 and 0.042, respectively). In the adjusted analysis, IL1-B and IL-13 remained associated with primary events, with *p* = 0.027 and *p* = 0.030, respectively, and IL1-B with total events (*p* = 0.022). In the logistic regression analysis, IL-5 independently predicted total events (*p* = 0.023). We found a significant association between tissue Doppler imaging variables and primary outcomes.

**Conclusion:**

Identifying IL-5, IL1-B and IL-13 as probable independent predictors of events at an early stage of Chagas disease is a significant milestone to the understanding of this pathology evolution. Also, several tissue Doppler parameters were important as prognosis predictors.

## Introduction

Chagas Disease (CD) represents a major public health issue in Latin America, affecting from six to seven million people in 21 countries.^1^^,^^2^ About 30 % of infected individuals develop chronic Chagas myocarditis,^3^ having a high risk of Heart Failure (HF), arrhythmias, and death. Most patients with the undetermined form of the disease remain asymptomatic for a long time, developing no heart disease.^4^^,^^5^ Individuals with positive serology and normal or abnormal Electrocardiogram (ECG) without HF constitute a large proportion of Chagas patients in populations in which this disease is endemic.

Electrocardiographic alterations, heart chamber dilation, thromboembolism, segmental or global contractility alterations, and ventricular arrhythmias indicate worse prognoses for HF and death.^5^, ^6^, ^7^, ^8^, ^9^, ^10^, ^11^, ^12^, ^13^ More modern techniques, such as cardiac magnetic resonance imaging and echocardiography with tissue Doppler and strain imaging can detect earlier changes in ventricular function.^14^, ^15^, ^16^, ^17^, ^18^, ^19^

Studies have evaluated tissue Doppler imaging via transthoracic echocardiography, showing evidence of its impact on the prognosis of patients with Chagas heart disease.^16^^,^^17^ Patients’ diastolic function in their left ventricle progressively worsens in all phases of the indeterminate form and early stages of CD, and tissue Doppler imaging configures the best marker of worsening diastolic function.^20^ Recent studies have shown that two-dimensional “strain” parameters offer independent factors for worse prognoses, (such as progression to HF or death) regardless of patients’ age, sex, and comorbidities.^18^^,^^19^^,^^21^, ^22^, ^23^, ^24^, ^25^, ^26^

On the other hand, chronic inflammation has a role in pathogenesis and progression to HF.^27^^,^^28^ The literature has described altered inflammation markers ‒such as pro-inflammatory cytokines (interleukins-1, −6, and −18 and TNF-alpha) and C-reactive protein ‒ in HF. Such markers are useful to stratify and find asymptomatic individuals at risk of heart failure. Some inflammation markers such as C-reactive protein and interleukin-6 seem to be related to the progression and more advanced stages of CD.^20^ Pro-inflammatory and modulating cytokines play an important role in tissue damage due to CD.^29^ Studies have suggested the anti-inflammatory role of IL-4, IL-10, IL-12, and IL-13 as critical for the clinical evolution of the disease and their possible correlation with the severity and therapeutic response of these patients.^30^^,^^31^ Moreover, low levels of IL-17 seem to be related to increased IL-12 and TNF levels and myocarditis and premature mortality.^32^

Nevertheless, patients in early stages of CD that received treatment with benznidazole developed higher IL-17 levels. These patients had better prognosis regarding biventricular function and functional class (based on NYHA classification), evincing the protective function of IL-17.^33^

This study aimed to evaluate the association of serum cytokine levels (IL-1, IL-2, IL-4, IL-5, IL-6, IL-7, IL-8, IL-10, IL-12p70, IL-13, IL-17, IFN-Y, TNF, CCL2/MCP-1, CCL4/MIP-1B, GCSF, and GMCSF) and the degree of impairment of left ventricular function (according to tissue Doppler imaging on an echocardiogram) with the progression of Chagas myocarditis in patients living with the indeterminate form of the disease and stages A or B1 of chronic heart disease (electrocardiographic alteration without changes according to the echocardiogram and no heart failure or arrhythmia), according to the 2nd Brazilian Consensus on Chagas Disease (2015).

## Methods

Patients who were aged from 18- to 60-years and who lived with CD were included into the convenience sample of this prospective longitudinal study. Data were collected from September 2011 to December 2012. Patients who had undergone no Transthoracic Echocardiogram (TTE) (*n* = 5) or had a diagnosis of HF (*n* = 1) and atrial fibrillation (*n* = 1) were excluded from this study. Collections were carried out in two cardiology reference outpatient clinics: Professor Edgard Santos University Hospital in Salvador/BA and in a cardiology reference center in the municipality of Itaberaba/BA. CD diagnosis was obtained by at least two positive serological tests (complement fixation, indirect hemagglutination, indirect immunofluorescence, or ELISA). Initially, 103 patients with the indeterminate form of CD and in stage A or B1 were chosen according to the classification of the 2nd Brazilian Consensus on Chagas Disease (2015): Stage A, ECG altered, TTE normal, no HF; Stage B1 (ECG altered, TTE altered and ejection fraction ≥ 45 %, no HF); Stage B2: Without HF, TTE with left ventricular ejection fraction < 45 %; Stage C: HF, altered TTE, compensated HF with or without arrhythmia; and Stage D: HF, altered TTE, and refractory HF with or without arrhythmia. To obtain this classification, the following examinations were performed in all patients at the beginning of this study: Chest X-Ray, 12-lead ECG, and TTE. Patients with a previous history of hospitalization for a heart condition or a documented severe arrhythmic event (such as ventricular tachycardia or second- or third-degree atrioventricular blocks) and those with pacemakers were excluded.

Echocardiograms were performed with a SAMSUNG Sonoview machine on all patients at baseline. Left ventricular end-diastolic volume, left ventricular end-systolic volume, and ejection fraction were obtained using Simpson’s biplanar rule. E and A waves and the E/A ratio were obtained from mitral inflow by pulsed Doppler; the velocities E’ (diastole) and S’ (Systole) were obtained by tissue Doppler imaging in the medial mitral (apical four chambers), lateral (apical four chambers), and inferolateral annuli (apical three chambers). The S’ wave of the lateral tricuspid annulus was collected from the apical four-chamber section. The E/E’ ratios of each mitral annulus, the mean E/E’ of the septal and lateral measurements and the mean of the S’ waves were calculated. All measurements of the cardiac structures were performed following the American Society of Echocardiography recommendations.

Blood was sampled from all patients. Random samples of their sera were stored for later analyses, dividing them into a subgroup of patients with disease progression and another without it. The following markers of inflammatory activity were measured in the FIOCRUZ laboratory: IL-1, IL-2, IL-4, IL-5, IL-6, IL-7, IL-8, IL-10, IL-12, IL-13, IL-17, IFN-Y, TNF, CCL2/MCP-1, CCL4/MIP-1B, GCSF, and GMCSF.

The following primary outcomes were chosen: progression to a group with greater cardiac involvement [according to the 2nd Brazilian Consensus on Chagas Disease (2015), development of Heart Failure with reduced Ejection Fraction (HFrEF), and death. In the case of patients who missed their medical appointments, the national registry of deceased persons was searched by a filter for municipalities in the state of Bahia.

The secondary outcomes included hospitalization for cardiac problems, pacemaker insertion, documented severe arrhythmic event (such as ventricular tachycardia or second- or third-degree atrioventricular blocks).

To investigate these patients’ clinical evolution, cardiovascular events, and death outcome, medical records and phone numbers were searched after about 10-years (2021). Included patients had to have a minimum of two years of follow-up or death at any time.

All participants signed an informed consent form. This study was approved by the Ethics Committee at the Professor Edgard Santos Hospital Complex (n° 77/10).

Statistical analyses were conducted using the Statistical Package for Social Sciences (v 18.0). Descriptive analyses were conducted by measures of central tendency and dispersion for continuous variables and by frequency measures for categorical variables. The Kolmogorov-Smirnov normality test was then applied to the continuous variables to define the comparison method to be used. Comparisons between groups were performed by the Student’s *t*-test for variables with almost normal distribution and by Pearson’s Chi-Squared test for dichotomous variables. An admissible alpha error of 0.05 was adopted for all statistical analyses. Analyses and survival curves were performed by the Kaplan-Meier method; an unadjusted Cox regression; and another regression adjusted for age, sex, phase of the disease, and treatment with benznidazole to evaluate predictors of primary and total events (primary or secondary outcomes). To estimate the Kaplan-Meier survival analyses and to obtain its curves, the groups with cytokine levels below or equal to the median for primary and total events and those with levels above it were compared. Univariate and multivariate analyses by binary logistic regression were performed to find the independent predictors of primary and total events. Multivariate analyses of survival by binary logistic and Cox regressions were performed for each cytokine with a *p* < 0.10 in the univariate analysis. The subsequent regressions were then controlled for age, gender, stage of the disease, and treatment with benznidazole. A *p* < 0.05 value was adopted as statistically significant.

## Results

We evaluated 61 patients in a prospective cohort from September 2011 to December 2021. Of these, 42 were women (68.9 %) with a mean age of 52.1-years and a median of 52.2-years. We found 16 patients with Right Bundle Branch Blocks (RBBB) (26.2 %), 11 with Left Anterior Fascicular Block (LAFB) (18 %), and six with both conditions (9.8 %). In total, 35 patients had systemic arterial hypertension, 4 had type 2 diabetes mellitus, and 4 had coronary artery disease. There was only one obese patient.

Twenty-six patients were in the indeterminate form of Chagas disease and 35 patients were in stage A or B1 of Chagas heart disease (Table 1).

**Table 1:** General characteristics of the patients according to the studied groups.

**Parameter**	**Category**	**N**	**%**
Sex	Female	42	68.9
	Male	19	31.1
Age	Below 54-years	38	62.3
	Above 54-years	23	36.1
Disease duration	<10-years since diagnosis	31	50.8
	≥10-years since diagnosis	27	44.3
Stage of the disease	Undetermined	26	42.6
	Heart disease (stage A)	35	57.4
RBBB	No	40	65.6
	Yes	16	26.2
LAFB	No	44	72.1
	Yes	11	18.0
RBBB + LAFB	No	55	90.2
	Yes	6	9.8
Hypertension		35	57.4
Diabetes mellitus		4	6.6
Coronary artery disease		4	6.6
Cardiovascular drugs	ACEI/ARB	34	55.7
	Calcium CB	14	23.0
	Statins	32	52.4
	Low socio-economic level	61	100

ACEI/ARB, Angiotensin-Converting Enzyme Inhibitor/Angiotensin Receptor Blocker; CB, Channel Blocker; LAFB, Left Anterior Fascicular Block; RBBB, Right Bundle Branch Block.

We found the following outcomes: 14 (23 %) primary events, with 5 deaths (8 %) and 14 cases of Heart Failure with Reduced Ejection Fraction (HFrEF) (23 %); 6 (10 %) secondary events, 4 required permanent pacemaker implantation (one presented sustained ventricular tachycardia with aborted sudden death) and 2 developed atrial fibrillation.

There were no significant differences between patients with outcomes and those without outcomes in relation to sex, age, BMI, socioeconomic status, education level, comorbidities, and use of cardiovascular medications. Furthermore, no cases suspected of Chagas disease reinfection were identified during clinical follow-up.

A large set of cytokines (a total of 16) was measured at baseline and compared between the groups with and without outcomes, according to means, standard deviations, confidence intervals, and p-values (Table 2).

**Table 2:** Serum cytokine level according to the presence or absence of a primary outcome.

**Primary outcome**						
**Cytokine**	**Outcome**	**Mean**	**Deviation**	**p-value**	**95% CI of the difference**	
					**Inferior**	**Superior**
IL1-B	Absent	32.20	36.79	0.48	−30.01	14.311
	Present	40.05	34.84	0.47	−30.12	14.42
IL-2	Absent	63.79	192.21	0.87	−96.60	113.63
	Present	55.27	66.09	0.80	−57.82	74.86
IL-4	Absent	33.33	23.51	0.87	−13.15	15.47
	Present	32.17	23.38	0.87	−13.65	15.96
IL-5	Absent	55.35	53.35	0.09	−63.53	4.57
	Present	84.84	64.07	0.13	−68.90	9.93
IL-6	Absent	75.62	83.99	0.97	−48.40	50.13
	Present	74.75	68.66	0.97	−44.51	46.24
IL-7	Absent	69.20	52.58	0.84	−34.44	28.05
	Present	72.40	46.41	0.83	−33.31	26.91
IL-8	Absent	58.27	64.87	0.30	−73.99	23.14
	Present	83.70	118.12	0.45	−95.51	44.66
IL-10	Absent	18.31	27.97	0.56	−10.79	19.86
	Present	13.78	10.10	0.36	−5.26	14.33
IL-12	Absent	142.90	170.91	0.66	−81.37	127.07
	Present	120.05	171.59	0.67	−85.61	131.31
IL-13	Absent	223.83	167.27	0.32	−48.14	145.85
	Present	174.97	126.64	0.25	−36.63	134.34
IL-17	Absent	156.49	181.97	0.56	−74.42	136.68
	Present	125.36	138.02	0.50	−61.98	124.24
GCSF	Absent	274.90	188.89155	0.52	−81.80	159.33
	Present	236.13	226.88	0.57	−100.78	178.32
GMCSF	Absent	60.28	164.99	0.31	−43.57	134.67
	Present	14.73	28.17	0.08	−5.01	96.11
MCP-1	Absent	72.48	65.14	0.53	−25.99	49.96
	Present	60.50	51.18	0.48	−22.20	46.17
MIP-1B	Absent	48.23	39.89	0.91	−26.57	23.67
	Present	49.68	45.65	0.92	−29.71	26.81
TNF-A	Absent	285.74	326.64	0.49	−129.06	266.95
	Present	216.79	319.10	0.49	−133.76	271.66
IFN-G	Absent	1906.64	1432.47	0.07	−416.99	1238.66
	Present	1495.81	1057.38	0.82	−308.32	1129.99

Unadjusted Cox survival analysis showed that IL1-B and IL-13 predicted primary and total events, with statistically significant p-values (Table 3).

**Table 3:** Unadjusted survival analysis by Cox regression for primary and total outcomes.

**Variables of interest**	**Primary outcome**				**Primary or secondary outcome**			
	**p-value**	**HR**	**95% CI**		**p-value**	**HR**	**95% CI**	
			**Inferior**	**Superior**			**Inferior**	**Superior**
IL1-B	0.015	1.029	1.005	1.053	0.013	1.026	1.005	1.047
IL-13	0.013	1.008	1.002	1.015	0.042	1.006	1.000	1.011
IL-5^a^	0.058	1.012(OR)	1.000	1.025	0.023	1.014 (OR)	1.001	1.027

a Binary logistic regression analysis. OR, Odds Ratio; HR, Hazard Ratio; CI, Confidence Interval.

**Fig. 1:**
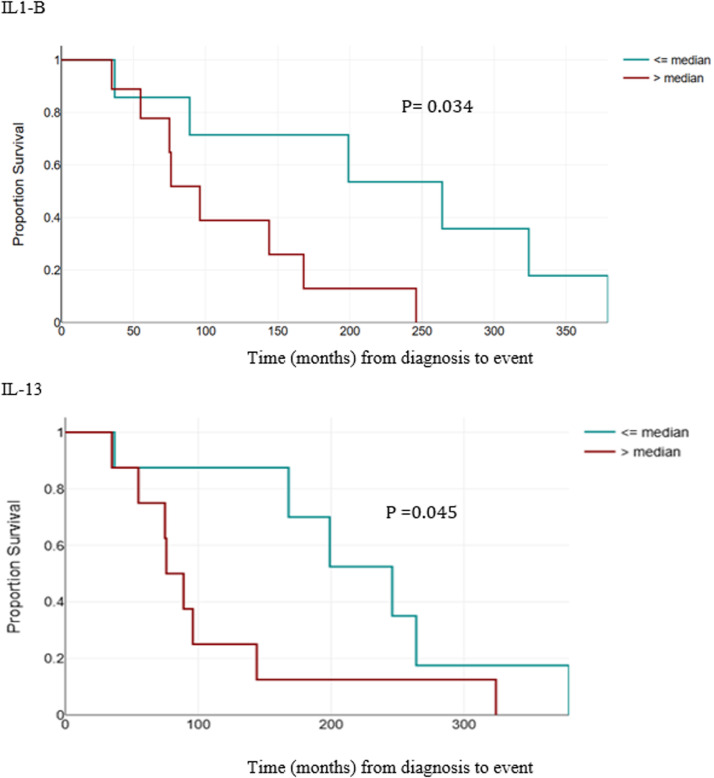
Kaplan-Meier Survival Curves for primary events: IL1-B and IL-13.

The analysis of survival adjusted for sex, age, evolutionary phase and treatment with benznidazole showed a positive association of IL1-B and IL-13 for primary events with a *p* = 0.027 and Hazard Ratios (HR = 1.031) and a *p* = 0.030 and HR = 1.008, respectively (Table 4 and Fig. 1). IL1-B also showed statistical significance for total outcomes, with *p* = 0.022. No statistical significance was found for primary and total outcomes for the other cytokines.

**Table 4:** Survival analysis for primary outcomes adjusted for sex, age, treatment, and entry group (follow-up phase).

**Variables of interest**	**Score**	**p-value**	**Hazard Ratio (HR)**	**95% CI**	
				**Inferior**	**Superior**
GCSF	0.643	0.423			
GMCSF	0.109	0.742			
IFN-G	0.242	0.623			
IL1-B	7.056	0.027	1.031	1.003	1.059
IL-2	0.484	0.486			
IL-4	0.025	0.875			
IL-5	0.045	0.833			
IL-6	2.406	0.121			
IL-7	0.182	0.670			
IL-8	0.064	0.800			
IL-10	0.082	0.774			
IL-12	0.047	0.828			
IL-13	7.073	0.030	1.008	1.001	1.016
IL-17	0.052	0.819			
MCP-1	0.281	0.596			
MIP-1B	0.021	0.884			
TNF-A	0.000	0.995			

Cox regression analysis, adjusted for sex, age, treatment, and subgroup (stage of the disease). OR, Odds Ratio; HR, Hazard Ratio; CI, Confidence Interval.

Regarding tissue Doppler alterations in undetermined CD or cardiac changes without HF, we observed a higher correlation with the primary outcomes E’ lateral (*p* = 0.018), E/E’ septal (*p* = 0.032), E/E’ lateral (*p* = 0.000), and mean E/E’ (*p* = 0.003) and with the secondary outcomes E’ septal (*p* = 0.032), E’lateral (*p* = 0.010), E/E’septal (*p* = 0.007), E/E’lateral (*p* = 0.000), mean E/E’ (*p* = 0.001), S’tricuspid (*p* = 0.015), S’ septal (*p* = 0.000), S’ lateral (*p* = 0.009), and mean S’ (*p* = 0.023).

We found no statistical significance between symptom variables and electrocardiographic alterations and the chosen primary and secondary outcomes.

## Discussion

This pioneering research analyzed a great number of cytokines, investigating their influence on patients’ clinical outcomes 10-years after its baseline evaluations. This research carefully dosed and quantified all chosen cytokines in the FIOCRUZ laboratory, generating a database to produce important information regarding the influence of cytokines on the progression of CD.

The use of serological markers, such as inflammatory cytokines, and cardiac imaging methods, such as tissue Doppler echocardiography, as we did in this research, provides strong tools to investigate progressive myocardial lesions in Chagas disease in two different ways: assessment of underlying pro-inflammatory activity and, on the other hand, subtle and subclinical changes in myocardial function.

The studied cytokines are involved in inflammatory processes, determining their intensity and subclinical damage. This process is slow in the chronic forms of CD, suffering the influence of, for example, gender and physical activity.^34^^,^^35^ In recent decades, studies have evaluated cytokines to understand the pathogenesis of the disease and to predict clinical outcomes.^36^, ^37^, ^38^, ^39^ However, only early markers of cardiomyopathy such as NT-ProBNP and troponin showed a correlation with unfavorable outcomes, even in the early phase of the disease. Cytokines might only rise in the bloodstream in more advanced stages of Chagas cardiomyopathy, impairing the analyses of its indeterminate form.^40^ Several studies have demonstrated increased levels of pro-inflammatory cytokines, including interferon gamma, TNF-alpha, IL-6, and IL-1β, in patients with Chagas heart disease; however, the independent prognostic value of these markers has been inconsistent across studies.^38^^,^^41^

All these studies, however, compared patients at different stages of Chagas disease, generally indeterminate phase versus advanced chronic heart disease with heart failure. This is not the best way to prove that a particular biomarker is a cause or effect. For example, it has been well demonstrated that blood levels of IL-6 rise in the presence of heart failure, even in Heart Failure with preserved Ejection Fraction (HFpEF), regardless of etiology and in non-Chagasic patients.

The importance of our study comes from the fact that it is the first longitudinal study to investigate the predictive value for major events such as death and heart failure of a wide variety of cytokines, over a period of 10-years, in patients without heart failure and without ventricular dysfunction.

We found IL-1β and IL-13 as predictors of primary and total outcomes and IL-5 as a probable independent predictor for total events. Interestingly, the activation pathways of IL-1β have been investigated experimentally, and the authors described that patient with the cardiac clinical form of CD showed an increase in IL-1β after stimulation.^20^ Conversely, IL-6 was not a predictor of events in our cohort. Caution is needed in interpreting these data, as the study does not have sufficient statistical power to invalidate the possibility that IL-6, TNF, or other cytokines may also be predictors of future events in an early stage of Chagas disease. This still needs to be proven in larger longitudinal studies.

Patients living with CD have a high prevalence of segmental wall alterations^42^ and diastolic dysfunction preceding systolic dysfunction. Tissue Doppler imagining and specially speckle tracking strain offer a non-invasive tool that can detect early, subclinical, myocardial changes. Many studies have shown a significant role of strain in Chagas disease. Tissue Doppler is a more accessible echocardiography method and has also been used to detect early myocardial changes in Chagas disease, even before ejection fraction. In our current study, we found several tissue Doppler variables (S', E') to be significantly reduced in the group that developed events.

New atrioventricular blocks and or intraventricular branch blocks (such as right bundle branch blocks associated or not with a left anterior fascicular block) and segmental alterations (evidenced by echocardiography imaging) characterize individuals who may develop ventricular dilation.^34^ We also analyzed the correlation between intraventricular alterations on the ECG and signs and symptoms, finding no statistical significance.

Chagas disease (a chronic infection caused by T. cruzi) presents a long low-grade inflammatory process with uncertain evolution, which makes it difficult to predict clinical outcomes, thus hindering the implementation of measures to reduce cardiovascular morbidity and mortality.

Great scientific interest lies in finding biomarkers regarding the CD progression. Our study collaborates with the literature finding IL1-B, IL-13 and IL-5 as probable predictors of events and reinforcing the important role of the study of myocardial function by tissue Doppler imaging.

### Study limitations and comments

A significant portion of the patients were lost to follow-up, which may have influenced our results. This is a relatively small cohort. However, it represents a rare group of patients with early-stage Chagas disease followed prospectively for approximately 10 years, which partially compensates for sample size limitations. The primary outcomes were identified in a significant proportion of patients (14/61, 23 %), which allowed for the analysis of the survival curve, an important aspect of the current study.

We are aware that the observed hazard ratios for cytokines are modest. Small effect sizes are expected in early-stage disease and multifactorial conditions such as Chagas cardiomyopathy. Furthermore, testing multiple cytokines without formal correction increases the risk of type I error. Given the exploratory nature of this study and the relatively small sample size, we chose not to apply strict corrections that could substantially increase type II error.

Mean cytokine levels did not greatly differ between groups in descriptive comparisons whereas survival analyses revealed significant associations. The rationale is that cytokines may influence the timing of events without producing large differences in baseline means. This reinforces the importance of longitudinal studies with survival analysis to demonstrate the true predictive value of variables.

No systematic investigation of smoking status and sedentary lifestyle was conducted. Obesity is related to inflammatory processes and worsening cardiovascular outcomes,^34^ however, there was only one obese patient in our cohort, and he belonged to the group that did not develop any adverse outcomes. Therefore, this was not significant in our study.

For all these reasons, our findings should be interpreted as hypothesis-generating and require confirmation in larger, independent, cohorts. We emphasize that these biomarkers are not proposed as standalone predictors but as components of multimodal risk stratification.

## Funding

This study was financed, in part, by the São Paulo Research Foundation (FAPESP), Brazil. Process Number #2022/02339-4 and #2024/17783-2.

## Data availability

The data that support the findings of this study are available from the corresponding author upon reasonable request.

## Conflicts of interest

The authors declare no conflicts of interest.
